# Surveillance of Live Poultry Markets for Low Pathogenic Avian Influenza Viruses in Guangxi Province, Southern China, from 2012–2015

**DOI:** 10.1038/s41598-017-17740-0

**Published:** 2017-12-14

**Authors:** Sisi Luo, Zhixun Xie, Zhiqin Xie, Liji Xie, Li Huang, Jiaoling Huang, Xianwen Deng, Tingting Zeng, Sheng Wang, Yanfang Zhang, Jiabo Liu

**Affiliations:** Guangxi Key Laboratory of Veterinary Biotechnology, Guangxi Veterinary Research Institute, 51 You Ai North Road, Nanning, Guangxi 530001 China

## Abstract

Infections with low pathogenic avian influenza viruses (LPAIVs) can be mild or asymptomatic in poultry; however, in humans, LPAIVs can cause severe infections and death, as demonstrated by the H7N9 and H10N8 human infection outbreaks in 2013 in China. In this study, we conducted an epidemiological survey of LPAIVs at live poultry markets (LPMs) in Guangxi Province, Southern China, which is near several Southeast Asian countries. From January 2012 to December 2015, we collected 3,813 swab samples from poultry at LPMs in Guangxi. Viral isolation, hemagglutination inhibition assay and viral sequencing were utilized to identify LPAIVs in the collected samples. Among the samples, 622 (16.3%) were positive for LPAIVs. Six subtypes (H1, H3, H4, H6, H9 and H11) were individually isolated and identified. Of these subtypes, H3, H6 and H9 were predominant in ducks, geese and chickens, respectively. Among the 622 positive samples, 160 (25.7%) contained more than one subtype, and H8, H10, H12, H13, and H16 were identified among them, which highlights the continuous need for enhanced surveillance of AIVs. These results provide detailed information regarding the epidemic situation of LPAIVs in the area, which can aid efforts to prevent and control AIV transmission in humans and animals.

## Introduction

Avian influenza viruses (AIVs) are enveloped viruses of the genus *Influenza A virus* in the family Orthomyxoviridae, and their genome consists of eight segments of single-stranded, negative-sense RNA. AIVs are classified into distinct subtypes based on the antigenicity of their hemagglutinin (HA) and neuraminidase (NA) proteins. To date, 18 HA subtypes (H1-H18) and 11 NA subtypes (N1-N11) have been identified. All of these subtypes were initially identified from avian species, with the exception of H17N10 and H18N11, which were recently found in bats^1,2^. According to Office International Des Epizooties (OIE) standards, AIVs are classified according to their level of virulence as either highly pathogenic AIVs (HPAIVs) or low pathogenic AIVs(LPAIVs)(http://www.oie.int/fileadmin/Home/eng/Health_standards/tahm/2.03.04_AI.pdf). A few H5 and H7 subtypes of AIV, such as H5N1 and H7N7, are virulent HPAIVs, leading to high morbidity and mortality in poultry. These subtypes are a public health security concern and cause severe economic losses in the poultry industry^3–5^. In contrast, LPAIVs strains, including the H1-H16 subtypes, typically cause mild disease or asymptomatic infections in poultry. Thus, LPAIVs have been largely neglected in global disease control programs. However, the genetic rearrangement and recombination of LPAIVs afford considerable opportunities for rapid and substantial viral changes through both antigenic shift and drift. Such changes may generate novel viruses with increased virulence that can pose substantial risks to poultry and human health.

In March 2013, H7N9, a novel influenza A virus causing human infection emerged in the Yangtze River Delta region with a fatality rate of approximately 30%. H7N9 quickly spread to more than 18 provinces and municipalities in China^6^. According to the Disease Outbreak News issued by the World Health Organization (WHO) on September 5,2017, 1,558 laboratory-confirmed cases of human infection with H7N9 virus have been reported since early 2013 (http://www.who.int/csr/don/5-september-2017-ah7n9-china/en/). During the same period, another novel emergent virus, H10N8, was detected in Jiangxi Province, China, resulting in 3 human infections and 2 deaths in December 2013^7^. Nevertheless, both the H7N9 and H10N8 strains showed few symptoms and caused silent out breaks in poultry and were therefore classified as LPAIVs; however, these strains retain the ability to infect humans and cause death. Unlike HPAIV outbreaks that are direct and obvious, LPAIVs, such as H7N9 and H10N8, are harder to detect in poultry until they cross the species barrier and directly infect humans. When reports of human deaths due to LPAIVs emerge, people stop purchasing live poultry in live poultry markets (LPMs), which results in large numbers of poultry products (e.g., chickens, ducks, geese and eggs) that are unable to be sold, thereby causing serious economic losses in the poultry industry. In addition, the genetic reassortment of LPAIVs is cause for concern. The recently emerged H7N9 and H10N8 AIVs are reassortant viruses, and the H9N2 LPAIV contributed six internal genes to the novel H7N9 and H10N8 viruses^8,9^. A naturally acquired human infection with the H6N1 virus was first documented in Taiwan in June 2013. Sequence analyses reveal that the human isolate (A/Taiwan/2/2013, human-H6N1) from the patient was highly homologous to chicken H6N1 virus isolates in Taiwan^10^. LPAIVs are closely related to human health and pose potential threats to public health security.

Southern China has been considered an influenza epicenter due to its favorable breeding grounds for the virus. Domestic poultry farming in Southern China occurs in high-density settings and in a free-range manner. Waterfowl, especially ducks and geese, occupy lakes and other abundant water resources in Guangxi, Southern China. These settings create environments where migratory birds and waterfowl are in close contact, sharing water, food, and habitat, thus contributing to the geographical spread of AIVs via long-distance migration through flyways. Thus, waterfowl species play an important role in AIV transmission and are regarded as a natural reservoir of AIVs^11^. Guangxi Province borders Vietnam, where complex epidemics of AIVs, including H5N1, continue to occur^12^. Furthermore, trade in LPMs, a traditional practice in this area, is considered a major source of AIV dissemination. In LPMs, birds from different sources can be housed together for several days, providing opportunities for influenza virus reassortment and the cross-species transfer of AIVs^13,14^. In LPMs, humans interact closely with poultry and potentially share influenza pathogens, which can result in the emergence of novel AIV variants.

Little is known about the infection landscape of LPAIVs in Guangxi. Our comprehensive investigation of LPAIV infection patterns and distribution in LPMs in this region from 2009 to 2011 revealed that the epidemiology of LPAIVs in poultry in Guangxi is complex, highlighting the need for further epidemiological study^15^. In the present work, we report on continuous surveillance data from January 2012 through December 2015 involving a relatively sizeable collection of swab samples from the oropharyngeal and cloacal regions of chickens, ducks, and geese from LPMs in Guangxi. We examined these samples for the presence of LPAIVs (except for H5 and H7 LPAIVs). The resulting epidemiological data offer novel insights for planning strategies to prevent LPAIV infections in humans and other animals.

## Results

### Isolation rates of LPAIVs in chickens, ducks and geese

We collected 3,813 swabs samples from 21 different LPMs in Guangxi Province from January 2012 to December 2015. The samples were used for the isolation and identification of LPAIVs. As shown in Table 1, among the 3,813 samples, 622 were identified as positive for LPAIVs, including 161 from chickens, 402 from ducks and 59 from geese. The total isolation rate was 16.3% (622/3,813, positive/sum), and the isolation rates in chickens, ducks and geese were 12.7% (161/1,267), 17.6% (402/2,280) and 22.2% (59/266), respectively. The rate of LPAIVs isolation was the highest from geese, followed by ducks and chickens, indicating that the isolation rate was higher in waterfowl. The numbers of positive samples and collected samples corresponding to the yearly isolation rates in chickens, ducks and geese from 2012 to 2015 are presented in Table 1. The isolation rate in ducks was slightly higher than the rate in chickens in 2012–2014 and was much higher than the rate in chickens in 2015. Compared with 2012, the isolation rates in both chickens and ducks increased to more than 20% in 2013 and then decreased to approximately 15% in 2014. The isolation rate of LPAIVs in chickens continued to decrease thereafter, reaching 8% in 2015, while that in ducks increased to 20.8%. The isolation rate in geese was highest (50.0%) in 2012. The isolation rate in geese decreased to 34.8% in 2013 and then to 9.8% in 2014, but it subsequently increased to 19.4% in 2015.

**Table 1:** LPAIVs isolated from chickens, ducks and geese from live poultry markets (LPMs) in Guangxi, Southern China, from January 2012 to December 2015.

Sample year	LPAIV isolation rate (%) Number of positive samples/total samples		
	Chickens	Ducks	Geese
2012	**7**.**5** (14/187)	**9**.**3** (33/356)	**50**.**0** (18/36)
2013	**21**.**6** (52/241)	**22**.**0** (95/431)	**34**.**8** (16/46)
2014	**14**.**8** (61/413)	**15**.**8** (117/740)	**9**.**8** (11/112)
2015	**8**.**0** (34/426)	**20**.**8** (157/753)	**19**.**4** (14/72)
Total (2012–2015)	**12**.**7** (161/1,267)	**17**.**6** (402/2,280)	**22**.**2** (59/266)
Grand total (all poultry samples 2012–2015)	**16**.**3** (**622/3**,**813**)		

**Figure 1:**
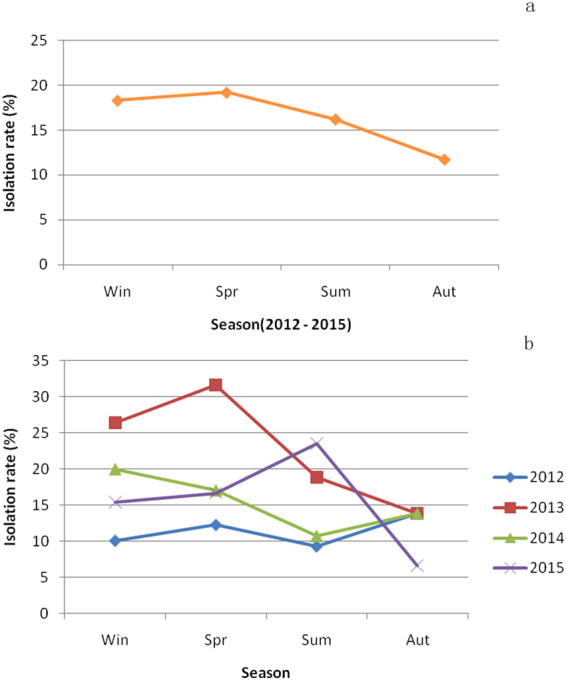
Relationship between season and isolation rates of LPAIVs in poultry from LPMs in Guangxi, Southern China, from 2012–2015. (**a**) Average isolation rates of each season over four years. (**b**) Isolation rates of each season annually.

The season average isolation rates in poultry, including chickens, ducks and geese, during the four years are shown in Fig. 1a. Higher isolation rates occurred in the spring and winter, and the isolation rates were relatively low in summer and autumn.

The season isolation rates are shown in Fig. 1b from 2012 to 2015. In general, the isolation rate in 2013 was higher than the rates in the other three years. The highest isolation rates from 2012 to 2015 were in autumn, spring, winter and summer, respectively. Generally, the isolation rates in spring and winter are higher than in summer and autumn, except for the rather high rates in autumn in 2012 and in summer in 2015.

### HA and NA subtypes identified in chickens, ducks and geese

More than six HA subtypes (H1, H3, H4, H6, H9, H11 and some that were unknown) were individually isolated and identified in poultry. The percentages of identified subtypes from poultry in the 622 positive samples and in the 3,813 collected samples are listed in Table 2. When considering only single infections, H9 was the most commonly isolated subtype; its percentages in all positive samples (24.1%, 150/622) and in collected samples (3.9%, 150/3,813) were the highest for the isolation of any single subtype (Table 2). The H9, H3 and H6 subtypes were the predominant LPAIVs identified in chickens, ducks and geese, respectively. It is equally important that 78 H4 isolates were identified, which were mainly from ducks.

**Table 2:** Number of infections of different HA subtypes of LPAIVs in chickens, ducks and geese.

Number	Subtype of infection	Number of cases				Percentages in positive samples (%)	Percentages in collected samples (%)
		Chickens	Ducks	Geese	Total		
1	H1	0	4	0	4	0.64	0.1
2	H3	7	**98**	2	107	17.2	2.8
3	H4	5	69	4	78	12.5	2.0
4	H6	10	70	**42**	122	19.6	3.2
5	H9	**99**	47	4	**150**	**24**.**1**	**3**.**9**
6	H11	0	1	0	1	0.16	0.026
7	Mixed infections	40	113	7	160	25.7	4.2
Total		161	402	59	622		

**Figure 2:**
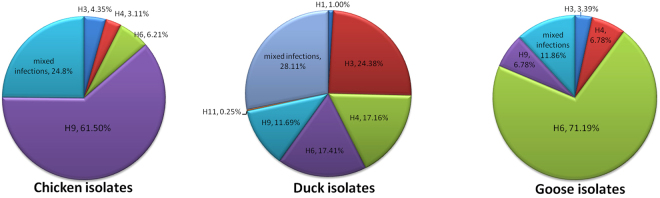
Distribution of HA subtypes among LPAIVs identified in poultry from LPMs in Guangxi, Southern China.

The distribution of the HA subtypes in poultry is shown in Fig. 2. The H9 subtype was predominant in chickens, representing 61.5% of the isolates, with the remaining three subtypes each representing less than 7%.The duck isolates were diverse, with six identified subtypes. H3 was the most frequent subtype (24.38%), followed by H6 (17.41%), H4 (17.16%) and H9 (11.69%).The least frequent were H1 and H11, which were isolated as single subtypes only in ducks. The goose isolates were less diverse than those of the ducks; the H6 subtype was predominant, with an isolation rate of 71.19%, followed by H4 and H9, at equal rates of 6.78%.

**Figure 3:**
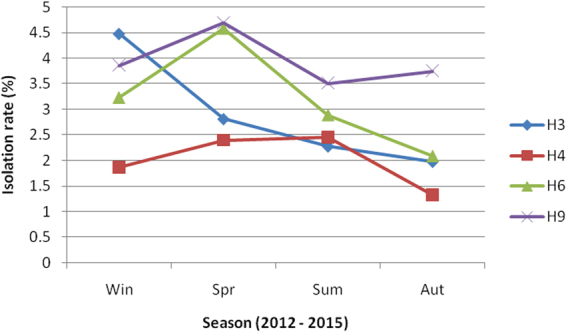
Relationship between season and isolation rates of the H3, H4, H6 and H9 subtypes in poultry from LPMs in Guangxi, Southern China, from 2012–2015.

The H1 and H11 subtypes were sporadic, while the H3, H4, H6 and H9 subtypes can be isolated in any season. Figure 3 shows the relationship between the main isolation subtypes and season. The highest isolation rate of the H3 subtype was in winter, and the H6 and H9 subtypes both peaked in spring. The HA and NA genes of the identified LPAIV isolates were sequenced and submitted to a BLAST search of the NCBI database. Three NA subtypes (N2, N6 and N8) were identified. The combinations of HA and NA subtypes identified in this study were H1N2, H3N2, H3N6, H4N2, H4N6, H4N8, H6N2, H6N6, H6N8, H9N2 and H11Nx (x has not been confirmed).

### Mixed infections by different HA subtypes

The rate of mixed infections (more than one subtype) among the identified LPAIVs was 25.7% (160/622). Among the 160 samples positive for mixed infections, 40 were from chickens, 113 were from ducks, and 7 were from geese (Table 2). Among the 161 LPAIV-positive chicken isolates, 40 presented with mixed infections, yielding a rate of 24.84%. The corresponding rates for ducks and geese were 28.11% and 11.86%, respectively (Fig. 2).These results suggest that various mixed infections were widespread, especially in ducks. Ducks likely play an important role in the reassortment events that lead to new AIV variants.

As shown in Tables 3 and 4, among the mixed infections, combinations of two HA subtypes, comprising 17 types, were the most common combination type in the three hosts (chickens, ducks and geese), representing 89.4% of the mixed infections (143/160). Combinations of three HA subtypes, comprising 7 types, were observed only in chickens and ducks, and combinations of more than three HA subtypes, comprising 3 types, were observed only in ducks. These findings indicate that mixed infections in ducks were complex.

**Table 3:** Number of mixed infections of different HA subtypes of LPAIVs in chickens, ducks and geese.

Combinations of mixed infection	Number of cases				Percentages in mixed infections (%)	Number of Types
	Chickens	Ducks	Geese	Total		
Two different HA subtypes	34	102	7	143	89.4	17
Three different HA subtypes	6	6	0	12	7.5	7
Four different HA subtypes	0	4	0	4	2.5	2
Six different HA subtypes	0	1	0	1	0.6	1
Total	40	113	7	160		27

**Table 4:** Mixed infections of different HA subtypes of LPAIVs in chickens, ducks and geese.

Number	Type of mixed infection (27 combinations)	Number of cases						
		Chickens	Ducks	Geese	Including H9(15 combinations)	Including H4(15 combinations)	Including H3(8 combinations)	Including H6(8 combinations)
1	H1 + H4	0	1	0		1		
2	H1 + H9	2	0	0	2			
3	H1 + H13	0	1	0				
4	H3 + H4	5	69	4		78	78	
5	H3 + H9	1	2	0	3		3	
6	H3 + H11	0	1	0			1	
7	H4 + H6	0	4	0		4		4
8	H4 + H9	11	11	0	22	22		
9	H4 + H13	0	5	0		5		
10	H6 + H9	1	1	0	2			2
11	H6 + H11	0	0	1				1
12	H6 + H13	2	4	1				7
13	H8 + H9	6	0	0	6			
14	H9 + H11	0	1	0	1			
15	H9 + H12	4	1	1	6			
16	H9 + H13	2	0	0	2			
17	H9 + H16	0	1	0	1			
18	H3 + H4 + H6	0	1	0		1	1	1
19	H3 + H4 + H9	0	1	0	1	1	1	
20	H3 + H4 + H11	0	1	0		1	1	
21	H4 + H6 + H9	1	0	0	1	1		1
22	H4 + H6 + H13	1	1	0		2		2
23	H4 + H8 + H9	3	2	0	5	5		
24	H4 + H9 + H16	1	0	0	1	1		
25	H1 + H4 + H6 + H10	0	2	0		2		2
26	H3 + H4 + H9 + H11	0	2	0	2	2	2	
27	H3 + H4 + H9 + H11 + H13 + H16	0	1	0	1	1	1	
Total		40	113	7	56	127	88	20
The infection rate					35.0% (56/160)	79.4% (127/160)	55.0% (88/160)	12.5% (20/160)

As shown in Table 4, the H3 + H4 subtype was the most common combination of mixed infection, representing 48.8% (78/160). Among the 78 strains of mixed infections with H3 + H4, 57 strains are from 2015, and 21 strains are from 2012–2014; therefore, the isolation of H3 + H4 increased sharply in 2015. The different HA subtype combinations yielded 27 different types of mixed infections. Among these 27 types, there were 15 combinations involving the H4 subtype, totaling 127 strains, and 79.4% (127/160) among the mixed infections. Notably, mixed infections of 3–6 HA subtypes all involved the H4 subtype, indicating that the H4 subtype plays a significant role in mixed infections. H9 subtype distributed in the 15 combinations and 56 strains, H3 subtype distributed in the 8 combinations and 88 strains, and H6 subtype distributed in the 8 combinations and 20 strains, suggesting that the H9, H3 and H6 subtypes were also important components in mixed infections. Generally, H4 was the most abundant subtype in mixed infections. The number of combinations and the infection rate of the H9 subtype were both higher than those for H6 but lower than those for the H4 subtype. The rate of mixed infection cases including H3 was higher than that including H9, but only distributed in the 8 combinations, which was lower than those for the H9 (15 combinations). The high rates of cases that included H3 or H4 were mainly due to high proportion of H3 + H4 in the mixed infection cases. In addition, H8, H10, H12, H13, and H16 were rare subtypes that were not isolated or identified among the single infections but were identified among the mixed infections.

## Discussion

AIVs are distributed worldwide, with a global impact on animal and human health. When a poultry bird, such as a duck, is infected with multiple subtypes of AIV, the eight gene segments from the different HA subtypes may exchange genetic information with each other, which can lead to the emergence of a novel AIV and a potentially unpredictable pathogenic strain that could infect humans. The outbreak of the highly pathogenic H5N1 strain in 1997 was the first evidence that AIVs had the capacity for direct transmission from avian species to humans^3^.The 2009 pandemic H1N1 influenza virus spread to humans in over 215 countries and caused hundreds of thousands of deaths^16^. Previously, it was thought that only the H5 and H7 subtypes posed a significant pandemic risk; however, it is now known that strains of other HA subtypes (e.g., H9, H6 and H10) can infect humans and have pandemic potential^17^. LPAIV surveillance is intrinsically difficult because most LPAIV strains do not cause symptoms or only cause mild symptoms in infected poultry, allowing the viruses to spread silently^18^. To address this obstacle, intensive surveillance must be conducted if newly emerged reassortant viruses are to be detected early.

Livestock breeding is extensive and booming in Guangxi, with many breeding farms and companies due to the subtropical climate. Many large-scale farms are located outside of the city and provide poultry production to meet strong and increasing consumer demand. Some of these products are sold to the adjacent Guangdong Province and Hong Kong. In some rural areas, chickens are freely raised on the mountainside, and ducks and geese range freely in pools, rivers and seaside areas. TheChina - ASEAN (The Association of Southeast Asian Nations) Exposition has convened in Nanning (the capital of Guangxi Province) annually since 2004, and people from different countries in Southeast Asia gather to discuss issues related to communication, culture and economy involved with the effective prevention and control of influenza viruses. The prevention and control of AIVs requires further attention and action in this region of the world. In addition, the seaside cities of Beihai and Fangcheng along the Beibu Gulf in Guangxi Province provide a good resting area for migrating wild birds. This area also has a humid environment, which may benefit the survival, growth and transmission of LPAIVs. To date, thirty cases of human H7N9 infection have been reported in Guangxi since the emergence of the first H7N9 human infection in early 2013. Among the thirty cases, there were three cases in 2014, no cases in 2015 and 2016, and 27 cases in 2017 (as of September 10) (http://www.gxhfpc.gov.cn/xzzc/2017/0221/34600.html and http://www.gxhfpc.gov.cn/gzdt/bt/2017/0616/38510.html). As a result, LPMs were closed and sterilized, and people refrained from purchasing poultry products, resulting in severe economic losses to the poultry industry.

In contrast to the results of our previous study^15^, the current work revealed that H9, not H3, was the most prevalent subtype, consistent with the current epidemic situation in China^17^. H9 is currently in widespread circulation, occurring mostly in combination with the N2 subtype. However, we did isolate a strain of H9N8 in 2009 (GenBank number: KF768214). H9N2 viruses have been found to possess increased fitness to escape immunization pressure and undergo antigen evolution and drift in chickens, leading to the prevalence of new genotypes^19^. H9N2 has also been reported to reassort with several other subtypes, including H5N1, H6N1 and H6N2^17^. Thus, H9N2 viruses are high on the list of candidates that could cause another human influenza pandemic. Presently, the H9N2 subtype is a primary focus of concern because it is the donor of six internal gene segments to the H7N9 and H10N8 novel reassortment avian influenza strains. It is well known that the H5N1 strain, which was responsible for the 1997 human influenza outbreak in Hong Kong, was found to contain internal genes derived from H9N2^20^. The crucial role of H9N2 viruses at the animal-human interface might be due to its wide host range, adaptation in both poultry and mammals, and extensive gene reassortment^17^. Our epidemiological survey revealed that the H9 subtype was an important component and widely distributed in 15 combinations (total 27 combinations) of the mixed infections. Among the chicken isolates, H9 viruses were the major isolation subtype. In our previous study^21^, the results of a receptor-binding analysis revealed that 16 of 17 strains of H9 Guangxi isolates bound to both α-2,6-linked glycans and α-2,3-linked glycans; only 1 strain bound solely to α-2,3-linked glycans. The binding of the human influenza virus receptor, Siaa2–6 Gal, suggests that chickens may serve as an intermediate host and may be the source of transmission of the influenza virus to humans.

In our previous research, H6N1, H6N2, H6N5, H6N6 and H6N8 and combinations of these five subtypes were identified during 2009–2011^15^.These five subtypes also circulated in Eastern China from 2002 to 2010^22^; however, in this study, only three H6 subtypes (H6N2, H6N6 and H6N8) were circulating in Guangxi Province between 2012 and 2015, with H6N2 and H6N6 being predominant. It was reported that the H6N2 and H6N6 viruses coexisted in LPMs in several provinces of Southern China between 2008 and 2011, and approximately 34% of the H6 isolates derived from LPMs have acquired the ability to bind to the human-like receptor^23^. The evidence suggests that the H6 subtype was widespread and combined with different NA subtypes. The H6 subtypes were common among the goose and duck isolates in this study, especially the goose isolates, representing much as 71.19% of the identified LPAIVs from geese; the reason for this requires further exploration. Currently, H6 AIVs have worldwide distribution, and strains of the virus have been detected in various animal species, suggesting that H6 has a broader host range than other subtypes^23^. Our previous research^24^ suggested that H6 subtype AIVs could be directly transmitted from ducks to pigeons. Many reports have revealed that H6 viruses can infect and be efficiently transmitted among mice and ferrets^25^ and that they circulate extensively and reassort frequently^24,26^. In addition, a serum antibody positive for H6 viruses has been found in poultry workers^27^, and a further study indicated that H6 seropositivity in human specimens in Southern China was significantly higher than in northern China^28^.

Six LPAIV subtypes were individually isolated in ducks, suggesting that ducks are an important host. In the duck isolates, the isolation rate of the H3 subtype AIV was the highest of all the subtypes identified, which emphasizes the importance of enhancing the surveillance of waterfowl-originating AIVs. Most of the H3 isolates matched with N2, while a few matched with N6 or N8. H3, particularly the H3N8 virus, is highly adaptive since it is found in multiple avian and mammal hosts. H3N8, which was first isolated in Miami in 1963, is the major cause of equine influenza; however, H3N8 viruses have not yet been isolated from humans^29^. The H3N8 and H3N2 isolates from LPMs were reported to have a close relationship with the H5N8 HPAIV circulating in Korea and the United States, suggesting that H3-like AIVs may contribute internal genes to the highly pathogenic H5N8 viruses^30^. In our previous research, a duck isolate named A/duck/Guangxi/175D12/2014(H3N6)^31^ showed reassortment events between H3 and the H5N6 and H7N2 influenza viruses. Genomic analyses of A/pigeon/Guangxi/020 P/2009 (H3N6)^32^ and A/goose/Guangxi/020 G/2009(H3N8)^33^ suggested that these viruses have undergone extensive reassortment with different AIV subtypes. Dequan Yang *et al*. previously reported that H3N2 isolates from LPMs and poultry slaughterhouses in Shanghai had reassorted with other AIVs, especially the H5 and H7 subtypes, probably in pigeons, domestic ducks, and wild birds^34^. An H3N2 isolate from Anhui, which also showed the highest sequence homology to the H7 AIVs, was presumed to be a reassortant of H3 and H7 AIVs^35^. H3 is the most ubiquitous subtype and has a wide host range, including humans, pigs, horses, dogs, cats, seals, poultry, and wild aquatic birds^29^. It was reported that an H3N2 isolate from duck could acquire the potential to infect humans after multiple infections in a pig population^36^. H3 causes the seasonal influenza that is widely found in humans. These findings highlight the need for further investigation, epidemiological surveillance, and genetic and evolutionary studies of H3-subtype AIVs.

H4 viruses also circulate widely throughout the world and are transmitted to mammalian hosts. Our study revealed that the isolation rate of H4 subtypes significantly increased from year to year. In the mixed infections, more than two subtypes included the H4 subtype, suggesting that H4 was highly infectious in combination with other AIV subtypes and had the advantage of increased potential for reassortment compared to other subtypes, thereby leading to the emergence and outbreak of novel subtype influenza viruses, which could threaten public health. We considered the H4 subtype worthy of particular research focus. We found that H4 viruses frequently matched with N2 and N6; however, some H4N8 viruses were also identified in our study. It was reported that H4N8 viruses isolated from shorebird contained a unique PB1 gene and caused severe respiratory disease in mice^37^. We chose some representative isolates of the H4 subtype from this study to experimentally infect specific-pathogen-free (SPF) chickens and did not observe clinical symptoms, which suggests low pathogenicity. Specific antibodies against H4-subtype AIVs were detected in sera from swine and from people working on a chicken farm^38,39^. Eight genes of H4N6 isolated from a duck farm were closely related to H4N6 viruses from LPMs in Shanghai, indicating a potential correlation between AIVs from LPMs and farms^40^. The H4 LPAIVs infected mice directly without prior adaptation^40,41^. The extensive reassortment of H4 AIVs is worrisome because it may produce hybrid viruses that can jump to humans and cause major public health issues, as occurred with the newly emerged H7N9 influenza virus^18^.Therefore, further investigations of the mechanisms of H4 AIV mutation and reassortment are important to prepare for potential pandemics.

Not only were the H9, H6, H3 and H4 the main subtypes in the single infections, but they were also important in the mixed infections. H8, H10, H12, H13 and H16 were also involved in mixed infections, but none of them have yet been isolated as a single infection. H8, H12, H13 and H16 were found infrequently compared with the other subtypes but emerged in mixed infections, revealing that they were active and in circulation in the area. These findings suggest a probable increasing trend and the need to monitor these subtypes. The H10 subtype existed in mixed infections and was not isolated alone in this survey; however, H10N8 has caused human deaths in Jiangxi Province in China. Epidemics of mixed infections are wide spread and complex, and the co-infection of different HA subtypes in the same bird presents a great risk of gene rearrangement, which can lead to novel subtypes of AIVs. Furthermore, mixed infections have increased viral diversity through reassortment between viruses from different sources.

In conclusion, we investigated the epidemiology of LPAIVs in poultry from LPMs in Guangxi Province, Southern China, an epicenter of avian flu. Our study demonstrates a high prevalence of LPAIVs in poultry in this area and highlights a need to further investigate the genetics and evolution of LPAIVs.

## Materials and Methods

### Ethics statement

The present study was approved and conducted in strict accordance with the recommendations in the guide for the care and use of routinely sampled animals in LPMs by the Animal Ethics Committee of the Guangxi Veterinary Research Institute. Biological samples were gently collected from healthy chickens, ducks and geese using aseptic cotton swabs. The birds were not anesthetized before sampling, and each sampled bird was observed for 30 min after sampling before being returned to its cage.

### Sample collection

The epidemiological surveillance of LPAIVs in poultry was conducted at 21randomly selected LPMs in Guangxi Province, Southern China. Among these LPMs, six were sampled continuously during the study period. We performed sampling once every week, and the six markets were sampled in turn. We collected 3,813 swab samples (an oropharyngeal swab and a cloacal swab from the same bird were placed into the same collection tube and counted as a single sample) from LPMs from January 2012 to December 2015. The swabs were maintained in 1 ml of transport medium containing antibiotics at 4 °C until arrival at the laboratory. The cotton swabs were repeatedly cleaned, wiped and discarded. The sample solutions were stored at −70 °C for viral isolation.

### Viral isolation and identification

Samples were thawed and centrifuged for 10 min at 3,000 × g at 4 °C, and 0.2 ml of the supernatant from each sample was inoculated into the allantoic cavity of 9- to 11-day-old SPF embryonated chicken eggs (Beijing Merial Biology Company, Beijing, China). The inoculated chicken embryos were incubated at 35 °C and observed daily. Allantoic fluid was harvested from the embryos that died within 120 h of inoculation. The chicken embryos that survived longer than 120 h post-inoculation were chilled at 4 °C, and the allantoic fluid was then harvested. The harvested allantoic fluid samples were then tested for hemagglutination (HA) using 1% suspensions of chicken erythrocytes. The hemagglutination inhibition (HI) assay was conducted to determine the HA subtypes of the HA-positive samples. Briefly, the HA-positive allantoic fluid samples were tested with sera of different HA subtypes (H1-H4, H6, H8-H16), Newcastle disease virus (NDV) and Egg drop syndrome (EDS) to determine the HA subtypes. The isolates selected for sequence analysis were biologically cloned by at least three rounds of limiting dilution in embryonated SPF eggs. HA subtypes were identified using the HI test and by sequencing. NA subtypes were determined by direct sequencing. Experiments using LPAIVs were conducted in biosafety level 2 laboratory or negative pressure biosafety laboratory facilities.

### Viral sequencing

Viral RNA was extracted from infected allantoic fluid using a Body Fluid Viral DNA/RNA Miniprep Kit (Axygen Biosciences, Hangzhou, China) according to the manufacturer’s instructions. cDNA was synthesized from viral RNA by reverse transcription with the 12-bp primer 5′-AGCAAAAGCAGG-3′. PCR was performed using specific primers as described in previous research to obtain the full-length HA and NA genes^42^. The PCR products were purified with the TaKaRa Agarose Gel DNA Purification Kit Ver. 2.0 (TaKaRa, Dalian, China) and sequenced by Invitrogen of Guangdong Co., Ltd. A BLAST search was performed to compare the confirmed sequences against the nucleotide sequences of all known HA and NA gene subtypes in the GenBank database, and the HA and NA subtypes of the isolates were determined and verified.
